# An Unusual Case of Anti-GBM Antibody Elevation in HIV-Associated Nephropathy

**DOI:** 10.1155/2014/956475

**Published:** 2014-06-03

**Authors:** Vinay Minocha, Raafat Makary, Andreea Poenariu

**Affiliations:** ^1^Department of Internal Medicine, Division of Nephrology, University of Florida College of Medicine-Jacksonville, 4th Floor, LRC 653-1 West 8th Street, No. L18, Jacksonville, FL 32209, USA; ^2^Department of Pathology, University of Florida College of Medicine-Jacksonville, 4th Floor, LRC 653-1 West 8th Street, No. L18, Jacksonville, FL 32209, USA

## Abstract

*Introduction.* The most commonly seen glomerular disease in HIV infected patients is HIV-associated nephropathy (HIVAN); however, a multitude of other nephropathies can occur in HIV infection with an almost equal cumulative frequency. We report an unusual case of a patient with clinical and histological evidence of HIVAN in which the diagnosis was initially confounded by the finding of an elevated serum anti-glomerular basement membrane (anti-GBM) antibody. *Case Presentation.* We present a case of a 27-year-old African American female with a history of schizophrenia, cocaine abuse, and HIV infection who upon admission to our hospital was found to have severe acute kidney injury requiring hemodialysis. Urine studies revealed nephrotic range proteinuria and a serological workup was positive for anti-GBM antibody elevation with a value of 91 units (normal: 0–20 units). A renal biopsy revealed HIVAN with no evidence of crescentic glomerulonephritis or anti-GBM disease. *Conclusion.* This case highlights the need for careful interpretation of anti-GBM antibody tests in HIV infected patients with kidney disease and, in particular, the need for biopsy confirmation of the diagnosis prior to starting therapy. More research is needed to study the prognostic correlation between the degree of anti-GBM antibody elevation in HIVAN and disease severity.

## 1. Introduction

Renal complications of HIV infection are becoming a major cause of morbidity and mortality in HIV infected patients. Of significance is the wide spectrum of glomerular lesions that is associated with renal dysfunction in this patient population [1]. The most commonly seen glomerular disease in HIV infected patients is HIV-associated nephropathy (HIVAN). It is not only the most common cause of chronic renal failure in this population, but it is also the third leading cause of end-stage renal disease among African-American males between the ages of 20 and 64 [2]. Other glomerular lesions can occur in HIV infection with an almost equal cumulative frequency and these include amyloidosis, minimal change disease, cryoglobulinemia, immunoglobulin A (IgA) nephropathy, membranous nephropathy, and membranoproliferative glomerulonephritis [3].

The multitude of nephropathies associated with HIV infection makes a definitive diagnosis of HIVAN challenging without a renal biopsy. We report an unusual case of a patient with clinical and histological evidence of HIVAN in which the clinical diagnosis was initially confounded by the finding of an elevated serum anti-glomerular basement membrane (anti-GBM) antibody. This case also highlights the implications of autoantibody elevation and in particular the possible mechanism of anti-GBM antibody elevation in disease pathogenesis.

## 2. Case Report

**Figure 1:**
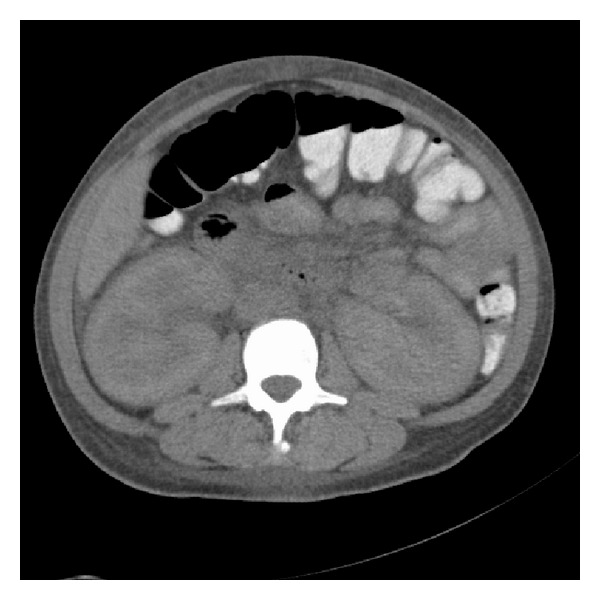
Computed tomography (CT) scan of the abdomen/pelvis without contrast revealing bilateral enlarged and edematous kidneys.

A 27-year-old African-American female presented to our hospital with a 3-day history of generalized cramping abdominal pain and nausea with vomiting. She denied any fevers, night sweats, weight changes, or urinary complaints. Her past medical history was significant for chronic paranoid schizophrenia, cocaine abuse, and HIV infection. She was not on highly active antiretroviral therapy (HAART) at the time of admission. Her vital signs included a heart rate of 120 beats/min and a blood pressure of 160/110 mmHg. Physical examination was remarkable for the presence of coarse crackles at the lung bases, periorbital and lower extremity edema, and mild abdominal distension. Laboratory studies revealed an elevated serum creatinine of 14.6 mg/dL, an elevated serum urea nitrogen of 86 mg/dL, and an elevated serum potassium of 6.9 mmol/L. Of significance, her renal function 4 months prior to this admission was normal with a creatinine of 0.8 mg/dL at that time. Urinalysis showed 2000 mg of protein per dL and urine microscopy revealed 6 red blood cells per high-power field and 22 white blood cells per high-power field but no dysmorphic red blood cells or casts. Other abnormal laboratory values on admission included a low serum albumin of 1.8 g/dL and a spot urine protein-to-creatinine ratio of 33.7 consistent with nephrotic range proteinuria. An ultrasound revealed enlarged kidneys with diffuse increased echogenicity and a computed tomography (CT) scan of the abdomen/pelvis without contrast revealed bilateral enlarged and edematous kidneys, scattered abdominal and pelvic ascites, and moderate soft tissue anasarca (Figure 1).

During the course of admission, the patient became tachypneic, had worsening oxygen saturations, and had to be intubated and mechanically ventilated for respiratory distress. Hemodialysis was emergently implemented after she became anuric and her renal function continued to deteriorate.

The patient's CD4 count at the time of admission was 350 cells/ul with a HIV RNA viral load of 1,790,000 copies/mL. Given the finding of nephrotic range proteinuria accompanied by a precipitous decline in renal function, a presumptive diagnosis of HIVAN was made and a serological workup was initiated to exclude other possible underlying etiologies. Serum protein electrophoresis, urine protein electrophoresis, anti-nuclear antibody, anti-double stranded DNA (anti-dsDNA) antibody, anti-streptolysin O, hepatitis serology, serum cryoglobulins, complement levels (C3, C4), and rapid plasma reagin tests were all negative. Her HbA1C was within normal limits at 5.8%. Serum testing was negative for cytoplasmic anti-neutrophil cytoplasmic antibodies (C-ANCA) and perinuclear anti-neutrophil cytoplasmic antibodies (P-ANCA). However, the serum was positive for anti-glomerular basement antibody (anti-GBM Ab) with a value of 91 units (normal: 0–20 units).

Although chest CT and bronchoscopy did not reveal diffuse alveolar hemorrhage that can be seen in anti-glomerular basement membrane antibody disease, the finding of an elevated anti-GBM antibody precluded a definitive diagnosis of HIVAN based on clinical context alone. A decision was therefore made to perform a CT-guided renal biopsy at this point.

**Figure 2:**
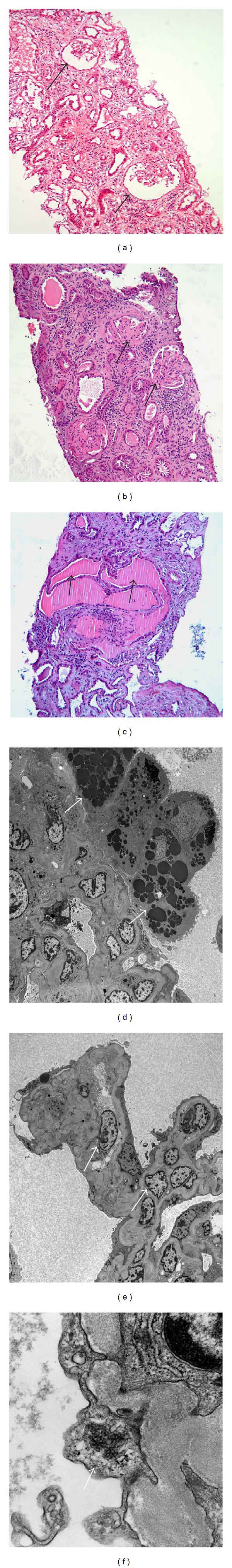
Light microscopy. (a) Shrunken glomerular tuft with dilated urinary space (arrows), (b) glomerular segmental sclerosis (arrows) with interstitial fibrosis, tubular degenerative/regenerative changes, and (c) tubular cystic dilatation (arrows) (H&E stain: (a) ×10, (b) ×10, and (c) ×20). Electron microscopy. (d) Several protein droplets in visceral Bowman's epithelium (arrows), (e) mesangial sclerosis with increased mesangial cellularity (arrows), and (f) dilated urinary space and tubuloreticular inclusions in tuft endothelium (arrow). No disruption or electron dense deposits in GBM (EM: (d) ×1900, (e) ×1900, and (f) ×30000).

**Figure 3:**
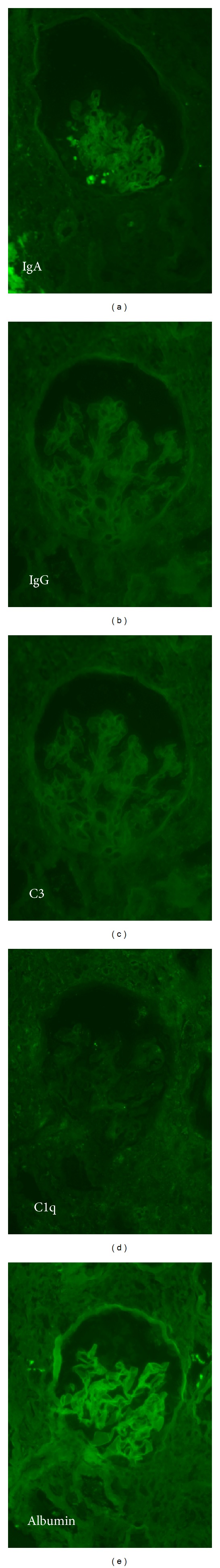
Immunofluorescence study negative for IgA, IgG, C3, and C1q (albumin control).

Light microscopy of the renal cortical tissue showed marked diffuse tubular degenerative and regenerative changes with interstitial fibrosis, focal microcystic tubular dilatation, and hyaline casts (Figures 2(a), 2(b), and 2(c)). Electron microscopy showed glomerular basement membrane thickening without disruption or electron dense deposits. Endothelial tubuloreticular inclusions most consistent with HIVAN were also visualized (Figures 2(d), 2(e), and 2(f)). The glomeruli showed focal segmental sclerosis with no evidence of crescentic glomerulonephritis and immunofluorescence revealed no specific immunoglobulin or complement deposits (Figure 3). Based on these pathologic findings, the final diagnosis was therefore HIVAN.

The patient continued to improve clinically and biochemically while receiving hemodialysis three times a week. She was eventually weaned from the ventilator and extubated. However, due to the patient's persistent dependence on hemodialysis throughout admission, it was concluded that the patient had end-stage renal disease (ESRD) and that she would require lifelong hemodialysis. She was also started on HAART therapy prior to discharge. At a 6-month follow-up visit, the patient remained compliant with HAART therapy and dialysis dependent.

## 3. Discussion

In the case presented, the patient had remarkably elevated levels of anti-GBM antibody without any clinical or histological evidence of Goodpasture syndrome or anti-glomerular basement membrane (anti-GBM) glomerulonephritis. Instead, a biopsy of the kidney was confirmatory for HIV-associated nephropathy. Previous studies have recognized this intriguing link between the asymptomatic elevation of anti-GBM and HIV infected patients with kidney disease, particularly HIV-associated nephropathy. However, the mechanism by which this occurs and its relevance to the disease process are still a matter of speculation.

HIVAN is defined by the presence of characteristic morphologic abnormalities found on renal biopsy. These features include the collapsing variant of focal segmental glomerulosclerosis (FSGS) combined with microcystic tubulointerstitial disease often with a modest cellular infiltrate [4]. As in this case, HIVAN typically presents with nephrotic syndrome, hypoalbuminemia, and large, echogenic kidneys by renal ultrasound. It is crucial that HIVAN be diagnosed in its early stages given its rapid progression to end-stage renal disease over weeks and months if untreated. Highly active antiretroviral therapy (HAART) has proven to be an effective treatment and it can prevent the progression of this disease [5].

On the other hand, anti-glomerular basement membrane antibody disease is defined by the presence of serum anti-GBM antibody and/or linear binding of IgG to GBM detected by direct immunofluorescence (IF) in a histological specimen in patients with crescentic rapidly progressive glomerulonephritis (RPGN). This disease can also cause pulmonary hemorrhage because of collagen epitope similarities in the basement membrane of both the lung and the kidney. Therefore anti-GBM disease can have two principal manifestations: renal-limited anti-GBM antibody disease and anti-GBM disease with pulmonary hemorrhage. The presence of pulmonary disease alone is very rare. As with HIVAN, the key to successful therapy and long term prognosis in anti-GBM disease is early diagnosis. Based on the results of several studies, standard treatment of anti-GBM antibody disease is a combination of plasmapheresis, prednisolone, and cyclophosphamide [6, 7].

In the case presented, the patient showed a subacute deterioration of kidney function without any form of treatment. She initially displayed no pulmonary manifestations such as hemoptysis, SOB, and coughing and had no systemic complaints such as malaise, fever, or hypertensive crisis typical of a vasculitis. The patient's urinary investigations revealed nephrotic range proteinuria and a lack of dysmorphic red cells without an active urinary sediment. Ultrasound studies revealed enlarged echogenic kidneys. All these clinical features pointed towards HIVAN as the underlying etiology of her deterioration in kidney function. However, the finding of an elevated anti-GBM antibody on initial workup confounded the diagnosis and highlighted the need for biopsy confirmation of the diagnosis.

Interestingly, the patient had a history of cocaine use and the association of anti-GBM disease with exposure to smoked and intranasal cocaine has been previously reported [8, 9]. Although our patient did not have anti-GBM disease, it has been postulated that cocaine can induce anti-GBM antibody formation by various pathogenetic mechanisms. Cocaine inhalation may cause tissue damage and probably expose pulmonary basement membrane antigens to subsequent antibody formation. Alternatively, cocaine may cause renal damage from cocaine-induced vasoconstriction and ischemia, causing release of basement membrane constituents and leading to anti-GBM antibody production [8].

Previous studies have shown that anti-GBM antibodies can be elevated in HIV infected patients who did not show clinical features of this condition. In a study conducted by Savige et al., plasma from 18 of 105 HIV infected individuals was found to have positive titers for anti-GBM antibodies. In this study and in contrast to the case presented, titers were borderline low in most of the patients studied. This study also found a significant correlation between the presence of anti-GBM antibodies and CD4 counts less than 400/ul [10]. This correlation was substantiated in the case presented as the patient had a CD4 count of 350 cells/ul when anti-GBM antibody titres were drawn. Of significance, this study also showed that, in addition to anti-GBM antibodies, ANA and ANCA can be commonly demonstrated in HIV infected individuals without any correlation between the presence of these antibodies and the corresponding autoimmune diseases [10].

A subsequent case report by Szczech et al. further highlighted the phenomenon of anti-GBM antibody elevation among three HIV infected patients with kidney disease. Although each patient had remarkably elevated levels of anti-GBM antibody, there was no evidence of anti-GBM antibody disease in two of the patients from whom biopsy specimens were taken and one patient without a biopsy specimen had a clinical course that was inconsistent with a diagnosis of anti-GBM antibody disease [11]. Although this phenomenon has been described, the exact mechanism by which it occurs and its relationship with kidney disease in HIV infected patients remain unclear.

In anti-GBM disease, antibodies are targeted against the type IV collagen present in glomerular and alveolar basement membranes. The specific epitope is the NC1 domain of the alpha 3 chain, but other antibodies against the alpha 5 and alpha 4 chains could also be detected [6]. In an animal model, it has been demonstrated that the alpha 3 chains from type IV collagen act as an immunogen in both forms, as dimer and as hexamer, but only antibodies induced by the former exhibit pathogenic capacity [12]. Autoreactive T-cells may be required for disease pathogenesis and it seems that regulatory CD4+ T-cells may play a role in regulation of the autoimmune response. A study done by Calderon et al. found antibodies to GBM in HIV negative patients with pneumonia caused by* Pneumocystis jirovecii *in the absence of kidney abnormalities. Based on this observation, the author suggested that* P. Jirovecii* could induce tissue injury and trigger an anti-GBM antibody form lacking pathogenic potential [13].

Although the exact mechanism has not been completely elucidated, HIV infects renal tubular epithelial cells through direct cell-cell transmission. In HIV-associated nephropathy (HIVAN), viral proteins can trigger the activation of cellular pathways that result in podocyte dysregulation characterized by increased proliferation, apoptosis, and dedifferentiation. Furthermore, HIVAN is also characterized by marked increase in apoptosis of renal tubular epithelial cells caused by viral protein expression [14]. It is therefore possible that the basement membrane damage or exposure that occurs as a byproduct of HIV infection of renal tubular epithelial cells and podocytes may trigger the production of nonpathogenic autoantibodies against specific epitopes on type IV collagen of the basement membrane. These nonnephritogenic forms of the antibody may be detected clinically by assays used for the measurement of anti-GBM antibodies.

The assay used by the laboratory for the measurement of anti-GBM antibodies in this case was the QUANTA Lite GBM (708740; INOVA, San Diego, CA, USA). This test is an enzyme-linked immunosorbent assay (ELISA) for the semiquantitative detection of glomerular basement membrane (GBM) IgG antibodies in human serum. Diluted sera are incubated in wells with GBM antigen bound to the walls thereby allowing any GBM antibodies present to bind to the immobilized antigen. A second incubation allows the enzyme labeled anti-human IgG to bind to any patient antibodies, which have become attached to the microwells. After washing away any unbound enzyme labeled anti-human IgG, the remaining enzyme activity is measured by adding a chromogenic substrate and measuring the intensity of the color that develops by spectrophotometry. This assay was found by Jaskowski et al. to have good sensitivity and specificity (100 and 92.3%, resp.) for the detection of anti-GBM antibodies [15]. It is possible that this assay may detect nonnephritogenic forms of the anti-GBM antibody.

Another possibility is that anti-GBM antibody production may be a secondary consequence of molecular mimicry, where there are shared peptide sequences between HIV particles and autoantigens [16]. By a similar mechanism, cross-reactivity with epitopes on HIV type 1 that mimic human self-proteins at a molecular level may cause a falsely positive anti-GBM antibody result when the conventional assay is used to detect these molecules in patients with HIVAN. A similar phenomenon can account for the fact that other autoantibodies such as ANA and ANCA are observed at a greater frequency in HIV infected patients compared to the general population without the accompanying autoimmune conditions [17]. Interestingly, autoantibody elevation is not unique to HIV infection. This phenomenon has also been seen in other viral infections such as hepatitis B virus, hepatitis C virus, cytomegalovirus, and Epstein-Barr virus [18].

Furthermore, the immune dysfunction that is associated with HIV infection is not limited to CD4+ T lymphocytes. It is a generalized process that also involves B-lymphocyte dysfunction secondary to defective T-lymphocyte regulation. As a result, polyclonal B-lymphocyte activation can occur and associated hypergammaglobulinemia has been described in HIV infected patients [19]. Pathogenetically, a comparison can be made between HIV infection and the polyclonal activation seen in systemic lupus erythematosus (SLE), rheumatoid arthritis, and myelodysplasia [10]. It is possible that the production of ineffective autoantibodies may be an inevitable consequence of this process.

## 4. Conclusion

This case highlights the need for careful interpretation of anti-GBM antibody tests in HIV infected patients with kidney disease and, in particular, the need for biopsy confirmation of the diagnosis prior to starting therapy. The significance of anti-GBM elevation in HIV-associated nephropathy and its relationship with disease pathogenesis and outcome remains unclear. More research needs to be done in order to elucidate whether autoantibody production may be an indicator of disease severity and its rapidity of progression. If a relationship between the degree of anti-GBM antibody elevation and progression of HIVAN is found, this autoantibody may be useful as a prognostic indicator for HIVAN in the future.

## References

[1] Wrone EM, Carey H, Reilly RF (1997). Glomerular lesions in HIV-infected patients. *Yale Journal of Biology and Medicine*.

[2] (2005). *US Renal Data System: USRDS 2005 Annual Data Report*.

[3] Wearne N, Swanepoel CR, Boulle A, Duffield MS, Rayner BL (2012). The spectrum of renal histologies seen in HIV with outcomes, prognostic indicators and clinical correlations. *Nephrology Dialysis Transplantation*.

[4] Ross MJ, Klotman PE, Winston JA (2000). HIV-associated nephropathy: case study and review of the literature. *AIDS Patient Care and STDs*.

[5] Elewa U, Sandri AM, Rizza SA, Fervenza FC (2011). Treatment of HIV-associated nephropathies. *Nephron Clinical Practice*.

[6] Lahmer T, Heemann U (2012). Anti-glomerular basement membrane antibody disease: a rare autoimmune disorder affecting the kidney and the lung. *Autoimmunity Reviews*.

[7] Fischer EG, Lager DJ (2006). Anti-glomerular basement membrane glomerulonephritis: a morphologic study of 80 cases. *American Journal of Clinical Pathology*.

[8] Peces R, Navascués RA, Baltar J, Seco M, Alvarez J (1999). Antiglomerular basement membrane antibody-mediated glomerulonephritis after intranasal cocaine use. *Nephron*.

[9] García-Rostán Y Pérez GM, García Bragado F, Puras Gil AM (1997). Pulmonary hemorrhage and antiglomerular basement membrane antibody-mediated glomerulonephritis after exposure to smoke cocaine (crack): a case report and review of the literature. *Pathology International*.

[10] Savige JA, Chang L, Horn S, Crowe SM (1994). Anti-nuclear, anti-neutrophil cytoplasmic and anti-glomerular basement membrane antibodies in HIV-infected individuals. *Autoimmunity*.

[11] Szczech LA, Anderson A, Ramers C (2006). The uncertain significance of anti-glomerular basement membrane antibody among HIV-infected persons with kidney disease. *American Journal of Kidney Diseases*.

[12] Kalluri R, Gattone VH, Noelken ME, Hudson BG (1994). The *α*3 chain of type IV collagen induces autoimmune Goodpasture syndrome. *Proceedings of the National Academy of Sciences of the United States of America*.

[13] Calderon EJ, Wichmann I, Varela JM (1997). Presence of glomerular basement membrane (GBM) antibodies in HIV-patients with Pneumocystis carinii pneumonia. *Clinical and Experimental Immunology*.

[14] Medapalli RK, He JC, Klotman PE (2011). HIV-associated nephropathy: pathogenesis. *Current Opinion in Nephrology and Hypertension*.

[15] Jaskowski TD, Martins TB, Litwin CM, Hill HR (2002). Comparison of four enzyme immunoassays for the detection of immunoglobulin G antibody against glomerular basement membrane. *Journal of Clinical Laboratory Analysis*.

[16] Golding H, Shearer GM, Hillman K (1989). Common epitope in human immunodeficiency virus (HIV) I-GP41 and HLA class II elicits immunosuppressive autoantibodies. Capable of contributing to immune dysfunction in HIV I-infected individuals. *Journal of Clinical Investigation*.

[17] Davenport A, Grant PJ (1990). False-positive autoantibodies in HIV infection. *The Lancet*.

[18] Weetman AP, Borysiewicz LK (1990). Viruses and autoimmunity. *Autoimmunity*.

[19] Lane HC, Masur H, Edgar LC, Whalen G, Rook AH, Fauci AS (1983). Abnormalities of B-cell activation and immunoregulation in patients with the acquired immunodeficiency syndrome. *The New England Journal of Medicine*.

